# Perceived food intolerance and irritable bowel syndrome in a population 3 years after a giardiasis-outbreak: a historical cohort study

**DOI:** 10.1186/s12876-015-0393-0

**Published:** 2015-11-19

**Authors:** Sverre Litleskare, Knut-Arne Wensaas, Geir Egil Eide, Kurt Hanevik, Gudrun Elise Kahrs, Nina Langeland, Guri Rortveit

**Affiliations:** Research Unit for General Practice, Uni Research Health, Kalfarveien 31, N-5018 Bergen, Norway; Centre for Clinical Research, Haukeland University Hospital, Bergen, Norway; National Centre for Tropical Infectious Diseases, Haukeland University Hospital, Bergen, Norway; Department of Clinical Nutrition, Haukeland University Hospital, Bergen, Norway; Department of Clinical Science, University of Bergen, Bergen, Norway; Department of Global Public Health and Primary Care, University of Bergen, Bergen, Norway

**Keywords:** Irritable bowel syndrome, Food intolerance, FODMAP, *Giardia lamblia*

## Abstract

**Background:**

Studies have shown an increased prevalence of irritable bowel syndrome (IBS) after acute gastroenteritis. Food as a precipitating and perpetuating factor in IBS has gained recent interest, but food intolerance following gastroenteritis is less investigated. The aims of this study were firstly, to compare perceived food intolerance in a group previously exposed to *Giardia lamblia* with a control group; secondly, to explore the relation with IBS status; and thirdly, to investigate associations with content of fermentable oligosaccharides, disaccharides, monosaccharides and polyols (FODMAP) in foods reported.

**Methods:**

This is a historical cohort study with mailed questionnaire to 1252 *Giardia* exposed and a control cohort matched by gender and age. Differences between groups were investigated using bivariate and multivariate analyses.

**Results:**

The questionnaire response rate in the exposed group was 65.3 % (817/1252) and in the control group 31.4 % (1128/3598). The adjusted odds ratio (OR) for perceived food intolerance for the exposed group was 2.00 with 95 % confidence interval (CI): 1.65 to 2.42, as compared with the control group. Perceived intolerance for dairy products was the most frequently reported intolerance, with an adjusted OR for the exposed of 1.95 (95 % CI: 1.51 to 2.51). Perceived intolerance for fatty foods, vegetables, fruit, cereals and alcohol was also significantly higher in the exposed group. The groups did not differ in perceived intolerance to spicy foods, coffee or soda. The association between exposure to *Giardia* infection and perceived food intolerance differed between the IBS group and the no-IBS group, but IBS was not a significant effect modifier for the association. Perceived intolerance for high FODMAP foods (adjusted OR 1.91) and low FODMAP foods (adjusted OR 1.55) was significantly associated with exposure status.

**Conclusion:**

Exposure to *Giardia* infection was associated with perceived food intolerance 3 years after giardiasis. IBS status did not alter the association between exposure status and perceived food intolerance. Perceived intolerance to high FODMAP foods and low FODMAP foods were both statistically significantly associated with exposure to *Giardia* infection.

**Electronic supplementary material:**

The online version of this article (doi:10.1186/s12876-015-0393-0) contains supplementary material, which is available to authorized users.

## Background

Gastroenteritis is a common condition around the globe, both sporadic cases and in larger outbreaks caused by contamination of drinking water or food. Post-infectious irritable bowel syndrome (PI-IBS) as a concept has been known for decades. Studies on patients with enteric infections have shown that 4–31 % develop PI-IBS [1]. Irritable bowel syndrome (IBS) has been described following infections caused by bacteria [1] (*Salmonella, E. coli, Shigella, Campylobacter*), virus [2] (*norovirus*) and parasites [3, 4] (*Giardia lamblia*). The mechanisms underlying the development of the disease are incompletely understood, and treatment options are currently the same as for sporadic irritable bowel syndrome [5].

Irritable bowel syndrome (IBS) is characterized by abdominal pain and/or discomfort related to alterations in bowel habits. It is a highly prevalent condition, and one recent meta-study found the pooled prevalence to be 11.2 % globally, varying according to country and the diagnostic criteria used [6]. It places a heavy burden on both the patient and the society, as measured by quality of life, use of health care resources, and work productivity [7]. The pathophysiologic mechanisms of the disease are yet to be fully understood. Current hypotheses include altered gastrointestinal motility [8], brain-gut-interactions [9] and visceral hypersensitivity [8]. There is a possible role of inflammation, post-infectious low-grade inflammation, genetic and immunologic factors, enteroendocrine cells and altered microbiota, but the results are inconsistent [9]. Patients with IBS often report that certain foods may trigger symptoms, and studies offer some support for this [10, 11]. Perceived food intolerance as a long-term complication after gastrointestinal infections is less investigated.

Effective treatment options for IBS are scarce. A dietary approach that has gained increased interest recently is the low FODMAP-diet. Fermentable oligosaccharides, disaccharides, monosaccharides and polyols (FODMAP) are a group of carbohydrates and sugar alcohols that share three functional properties: They are poorly absorbed in the small intestine, they are osmotically active molecules because of their small size, and they are rapidly fermented by bacteria [12]. Because of these characteristics there is a possibility that they may worsen symptoms in IBS patients, particularly in the presence of visceral hypersensitivity. Studies have shown that IBS patients may benefit from a diet low in FODMAP [13–17], but there has been some critique of the methodology in these studies, including the fact that no studies have been conducted on unselected patients from primary care [18]. There is a need for studies that elaborate the role of diet in IBS.

In the autumn of 2004 there was a large outbreak of giardiasis in the city of Bergen on the western coast of Norway. In a controlled follow-up study with nearly 2,000 participants in 2007 it was found that the group subject to *Giardia* infection 3 years prior had a significantly higher prevalence of IBS (46 %) than the control group (14 %) [4].

The aims of the current study were firstly, to compare the prevalence of perceived food intolerance in the two groups; secondly, to explore how this was related to the IBS status in the two groups; and thirdly, to investigate any associations with FODMAP content.

## Methods

### Participants

During the outbreak of giardiasis in Bergen in 2004, 1252 patients who had infection verified by detection of *Giardia lamblia* in their stools were included and comprised the *Giardia* exposed group. A 2:1 matched control cohort was established by sampling two people of the same age and gender for each exposed patient from the entire population of Bergen. Four controls were excluded due to giardiasis during the outbreak, as self-reported in the study questionnaire. The questionnaires were sent by mail in October 2007, and again one month later to non-respondents. Because of a low response rate in the primary control group, the questionnaire was mailed to an additional 1094 controls in May 2008. Details about the study population have been published previously [4].

### Variables

The primary outcome in this report is the respondents’ self-reported reactions to food, hereafter referred to as perceived food intolerance in line with previous literature [11, 19]. All respondents were asked the following question (Question A): “Do certain types of food give you abdominal symptoms?” Possible answers were: None, light, moderate and severe. For some analyses, these answers were further dichotomized into none vs. light, moderate or severe. Question A was followed by an open-ended question (Question B): “If you react (to food), to what kind is that?” For respondents who answered “no symptoms” or had a missing answer to Question A but still gave an affirmative response about specific types of food causing symptoms, the response was reclassified as “light”. The unmodified “light” category included 582 respondents, whereas the modified one included 606.

The responses to Question B were categorized in accordance with categories used in previous studies on IBS and food intolerance [10, 11, 19, 20], and are hereafter referred to as food categories. A selection of these food categories was further analysed. Reported foods were also categorized based on assumed content of FODMAP (high or low), hereafter referred to as high FODMAP foods and low FODMAP foods. FODMAP content of the foods reported was assessed using a mobile app developed by a research team at the Department of Gastroenterology, Central Clinical School, Monash University, Melbourne, Victoria, Australia. This reference tool was developed on the basis of results from food quantification studies [21–23]. The coding was also discussed between the first author and the clinical dietician in the research team (GK). A total of 971 respondents answered Question B. Responses that were coded as high FODMAP foods include vegetable, cakes, wheat, milk, apple, pear, prunes, dried fruit, and onions. Examples of responses coded as low FODMAP foods were sugar, cocoa, oil, rice, berries, strawberry, alcohol, soda, and banana. Foods where assumed FODMAP content could not be decided were categorized as “uncertain FODMAP.” This category was not further analysed. High FODMAP foods were classified according to what sub-group of FODMAP (oligosaccharides, fructose, polyols or lactose) they might contain. Details concerning the coding of the variables were accounted for in a codebook (available upon request).

Exposure in our study was defined as laboratory confirmed *Giardia lamblia* infection in 2004.

In the current study we wanted to investigate if there was an effect modification by IBS on the association between *Giardia* exposure and perceived food intolerance. IBS was defined according to the Rome III criteria. A detailed description of this part of the questionnaire and the translation procedure has previously been published [4].

Demographic information obtained was age (recorded as a continuous variable, categorized to 20-year groups) gender, marital status (four categories), level of education (three categories), main occupation (originally eight categories, reduced to four in the analyses) and status as a student or not in the autumn of 2004. Mean age was calculated before age was categorized to 20-year groups.

### Statistical analyses

Pearson’s chi square test (exact) was performed on differences between proportions. Results are reported as percentages with p-values for differences, or as unadjusted and adjusted odds ratios (OR) with 95 % confidence intervals (CI). Confounding and effect modification were evaluated with logistic regression modelling, and in stratified crosstabs with Breslow-Day test. Confounders evaluated were status as student or not in 2004, age, gender, work, income and level of education. All analyses of the primary outcomes were adjusted for gender and age. Effect modification by IBS on the association between exposure and perceived food intolerance was investigated by stratified cross tabulation and Breslow-Day test.

All tests were two-sided. The level of significance was 0.05. The data was analysed using the statistical software SPSS version 22.

### Ethical approval

This study has been approved by the Regional Committee for Medical and Health Research Ethics (project 150.07) and by the Ombudsman for Privacy in Research, Norwegian Social Science Data Services (project 17014). Respondents were informed that by completing and submitting the questionnaire, they consented to participate in the study.

## Results

The questionnaire response rate was 65.3 % (817/1252) among the *Giardia* exposed and 31.4 % (1128/3598) among controls, giving a total response rate of 40.1 % (1945/4850). Respondents were older than non-respondents (mean age 36.1 vs. 32.9 years, *p* < 0.001). There also were a higher proportion of females among respondents (65.7 % vs. 55.8 %, *p* < 0.001), as previously reported [4, 24]. Out of 1945 participants in total, 1875 (96.4 %) could be classified as having IBS or not. As expected from the matched design, the two groups did not differ with respect to gender and age. Further characteristics of respondents in the exposed and the control group are shown in Table 1.

**Table 1:** Characteristics of 817 *Giardia* exposed and 1128 controls in Bergen, Norway 3 years after outbreak of *Giardia*-epidemic in 2004

Characteristic	Exposed *N* = 817		Controls *N* = 1128		P
	N	%	N	%	
Age groups, years					0.107
0–19	39	4.8	36	3.2	
20–39	526	64.4	736	65.2	
40–59	187	22.9	276	24.5	
60–79	56	6.9	76	6.7	
80–99	9	1.1	4	0.4	
Females	540	66.1	738	65.4	0.772
Marital Status					0.003
Single	271	33.5	293	26.1	
Married	497	61.4	778	69.3	
Divorced/separated	33	4.1	41	3.7	
Widow/widower	9	1.1	11	1.0	
Education					0.004
Primary school	37	4.7	59	5.3	
Secondary school	169	21.3	308	27.7	
University	587	74.0	746	67.0	
Source of income					<0.001
Working	576	71.1	881	78.7	
Out of Work	70	8.6	96	8.6	
Student	137	16.9	121	10.8	
Other	27	3.3	22	2.0	
Student autumn 2004					<0.001
No	503	62.7	842	75.8	
Yes, full time	261	32.5	229	20.6	
Yes, part time	38	4.7	40	3.6	
IBS	355	46.1	155	14.0	<0.001

*Abbreviations: IBS* Irritable Bowel Syndrome, *P* P-value from Pearson’s chi square (exact)

Question A (if, and to what degree, food was perceived to cause abdominal symptoms) was answered by 95.8 % of the respondents (1864/1945). An additional 19 cases were missing from the combined analyses on IBS status and symptoms (Table 2). Among *Giardia* exposed 63.9 % reported perceived food intolerance as compared to 47.6 % in the control group, giving an adjusted odds ratio of 2.00 (95 % CI: 1.65 to 2.42). When stratifying according to IBS status, there were no significant differences between the exposed and controls in the IBS-group regarding perceived food intolerance. Within the no-IBS group the prevalence of perceived food intolerance was higher among the exposed (49 %) than the controls (42.3 %) (adjusted OR: 1.36, 95 % CI: 1.07 to 1.72). However, the Breslow-Day test for effect modification was negative, meaning that the difference in odds ratio between the IBS and the no-IBS group was not statistically significant.

**Table 2:** Perceived food intolerance in *Giardia* exposed (*n* = 764) and a control group (*n* = 1100) 3 years after an outbreak of giardiasis in Bergen, Norway, 2004

		Perceived food intolerance^a^														
Group		No		Yes^b^						Severity of perceived food intolerance						
						Unadjusted		Adjusted^c^		Light		Moderate		Severe		P
	N	n	%	n	%	OR	95 % CI	OR	95 % CI	n	%	n	%	n	%	
*Exposure status*																
Exposed	764	276	36.1	488	63.9	1.94	1.61 to 2.35	2.00	1.65 to 2.42	238	31.2	168	22.0	82	10.7	<0.001
Control	1100	576	52.4	524	47.6					368	33.5	116	10.5	40	3.6	
*IBS status*																
IBS	501	98	19.6	403	80.4	5.16	4.04 to 6.60	5.03	3.93 to 6.45	147	29.3	167	33.3	89	17.8	<0.001
No-IBS	1344	748	55.7	596	44.3					451	33.6	113	8.4	32	2.4	
*Within IBS*																
Exposed	348	65	18.7	283	81.3	1.20^d^	0.75 to 1.92	1.25	0.78 to 2.01	105	30.2	111	31.9	67	19.3	0.418
Control	153	33	21.6	120	78.4					42	27.5	56	36.6	22	14.4	
*Within No-IBS*																
Exposed	406	207	51.0	199	49.0	1.31^d^	1.04 to 1.66	1.36	1.07 to 1.72	130	32.0	55	13.5	14	3.4	<0.001
Control	938	541	57.7	397	42.3					321	34.2	58	6.2	18	1.9	

*Abbreviations: IBS* irritable bowel syndrome, *P* p-value from Pearson’s chi square test (exact), *CI* confidence interval, *OR* odds ratio

^a^The question pertaining to these categories was: “Do certain types of food give you abdominal symptoms?” with four alternatives: none, light, moderate, severe

^b^Four level response variable dichotomized to no (none) vs. yes (light, moderate or severe)

^c^Adjusted for gender and age

^d^The Breslis reasonably high, howeverow-Day test was non-significant

Question B (types of food perceived to cause symptoms) was answered by 49.9 % (971/1945) (Table 3). Dairy products was the most frequently reported food category, and was reported significantly more often in the exposed group than among controls with an adjusted OR of 1.95 (95 % CI: 1.51 to 2.51). Food categories created based on the responses were (in order of descending frequency given for the total study population in parentheses): Dairy products (292), spicy foods (256), vegetables (232), cereals (227), milk (163), fruit (120), alcohol (109), meats (93), coffee (91), fatty foods (75), wheat (73), unclassified (46), sweets (45), soda (40), sugar (33), dinners (29), gravy/dressing (28), beer (26), chocolate (25), juice (25), eggs (23), baked goods (22), fish (21), shellfish (21), yeast products (18), smoked food (17), nuts (17), processed food (15), tomato/tomato products (15), fruit juice (14), gluten (11), fibre (8), tea (8), salted food (8), soy (3).

**Table 3:** Perceived food intolerance according to food categories and FODMAP content in 817 *Giardia* exposed and 1128 controls three years after an outbreak of giardiasis in Bergen, Norway, 2004

	Exposed *N* = 817		Controls *N* = 1128		Unadjusted		Adjusted^b^	
Food categories^a^	n	%	n	%	OR^c^	95 % CI	OR^c^	95 % CI
*Food categories*								
Dairy products	163	20.0	129	11.4	**1.93**	**1.50 to 2.48**	**1.95**	**1.51 to 2.51**
Spicy foods	119	14.6	137	12.1	1.23	0.95 to 1.61	1.25	0.96 to 1.63
Fatty foods	48	5.9	27	2.4	**2.55**	**1.57 to 4.12**	**2.63**	**1.62 to 4.26**
Vegetables	118	14.4	114	10.1	**1.50**	**1.14 to 1.98**	**1.56**	**1.18 to 2.06**
Fruit	75	9.2	45	4.0	**2.43**	**1.66 to 3.56**	**2.45**	**1.67 to 3.60**
Cereals	128	15.7	99	8.8	**1.93**	**1.46 to 2.55**	**1.98**	**1.49 to 2.62**
Alcohol	66	8.1	43	3.8	**2.22**	**1.49 to 3.29**	**2.29**	**1.54 to 3.40**
Coffee	39	4.8	52	4.6	1.04	0.68 to 1.59	1.05	0.68 to 1.61
Soda	17	2.1	23	2.0	1.02	0.54 to 1.92	1.03	0.55 to 1.94
*FODMAP Content* ^*d*^								
High FODMAP	308	37.7	277	24.6	**1.86**	**1.53 to 2.26**	**1.91**	**1.57 to 2.33**
Low FODMAP	230	28.2	231	20.5	**1.52**	**1.23 to 1.88**	**1.55**	**1.26 to 1.92**
*FODMAP subtype*								
Oligosaccharides	190	23.3	187	16.6	**1.53**	**1.22 to 1.91**	**1.58**	**1.25 to 1.99**
Lactose	156	19.1	128	11.3	**1.84**	**1.43 to 2.38**	**1.86**	**1.44 to 2.40**
Polyols	78	9.5	45	4.0	**2.54**	**1.74 to 3.71**	**2.57**	**1.76 to 3.77**
Fructose	71	8.7	39	3.5	**2.66**	**1.78 to 3.97**	**2.69**	**1.80 to 4.02**

*Abbreviations: FODMAP* fermentable oligo-, di- and monosaccharides and polyols; *IBS* irritable bowel syndrome; *CI* confidence interval; *OR* Odds ratio.

^a^The question pertaining to these categories was: “If you react (to food), to what kind is that?”

^b^Adjusted for gender and age

^c^Statistically significant ORs are presented in **bold** font

^d^Assumed FODMAP content of the response(s) to the open-ended question about food

Perceived intolerance to high FODMAP foods was reported more often in the exposed group compared to the control group with an adjusted OR of 1.91 (95 % CI: 1.57 to 2.33), as was intolerance to low FODMAP foods, with an adjusted OR of 1.55 (95 % CI: 1.26 to 1.92). A total of 585 respondents reported intolerance to high FODMAP foods, 461 reported intolerance to low FODMAP foods, and 528 respondents reported intolerance to foods where FODMAP content could not be ascertained. The ORs for high FODMAP foods were somewhat larger than the ORs for low FODMAP foods (Tables 3 and 4). Since these categories were not mutually exclusive, there was no direct way to test the potential differences in strength between these associations statistically.

**Table 4:** Comparison of 770 *Giardia* exposed and 1105 controls stratified to IBS status, on perceived food intolerance according to food categories and FODMAP content 3 years after outbreak of a Giardia-epidemic in Bergen, Norway, 2004

	IBS N = 510								No-IBS N = 1365							
	Exposed *N* = 355		Controls *N* = 155		Unadjusted		Adjusted^b^		Exposed *N* = 415		Controls *N* = 950		Unadjusted		Adjusted^b^	
Food categories^*a*^	n	%	n	%	OR	95% CI	OR	95 % CI	n	%	n	%	OR	95 % CI	OR	95 % CI
*Food Categories*																
Dairy products	96	27.0	38	24.5	1.14	0.74 to 1.76	1.18	0.76 to 1.84	64	15.4	90	9.5	1.74	1.24 to 2.46	1.78	1.26 to 2.53
Spicy foods	69	19.4	26	16.8	1.20	0.73 to 1.97	1.24	0.75 to 2.04	48	11.6	109	11.5	1.01	0.70 to 1.45	1.04	0.72 to 1.49
Fatty foods	31	8.7	8	5.2	1.76	0.79 to 3.92	1.79	0.80 to 4.02	16	3.9	18	1.9	2.08	1.05 to 4.11	2.19	1.10 to 4.34
Vegetables	79	22.3	24	15.5	1.56	0.95 to 2.58	1.69	1.02 to 2.81	39	9.4	89	9.4	1.00	0.68 to 1.49	1.08	0.72 to 1.60
Fruit	49	13.8	12	7.7	1.91	0.99 to 3.70	2.04	1.05 to 3.97	25	6.0	31	3.3	1.90	1.11 to 3.26	1.96	1.14 to 3.38
Cereals	91	25.6	36	23.2	1.14	0.73 to 1.77	1.21	0.77 to 1.89	36	8.7	63	6.6	1.34	0.87 to 2.05	1.41	0.91 to 2.16
Alcohol	43	12.1	8	5.2	2.53	1.16 to 5.52	2.57	1.18 to 5.61	22	5.3	34	3.6	1.51	0.87 to 2.61	1.55	0.89 to 2.69
Coffee	23	6.5	12	7.7	0.83	0.40 to 1.70	0.84	0.40 to 1.73	16	3.9	40	4.2	0.91	0.51 to 1.65	0.92	0.51 to 1.66
Soda	9	2.5	6	3.9	0.65	0.23 to 1.85	0.67	0.23 to 1.91	7	1.7	17	1.8	0.94	0.39 to 2.29	0.96	0.39 to 2.34
*FODMAP Content* ^*c*^																
High FODMAP	186	52.4	74	47.7	1.21	0.83 to 1.76	1.27	0.87 to 1.87	116	28.0	201	21.2	1.45	1.11 to 1.88	1.51	1.15 to 1.98
Low FODMAP	145	40.8	57	36.8	1.19	0.81 to 1.75	1.22	0.83 to 1.80	83	20.0	171	18.0	1.14	0.85 to 1.53	1.17	0.88 to 1.58
*FODMAP subtype*																
Oligosaccharides	131	36.9	51	32.9	1.19	0.80 to 1.78	1.29	0.86 to 1.94	58	14.0	135	14.2	0.98	0.70 to 1.37	1.04	0.74 to 1.46
Lactose	93	26.2	39	25.2	1.06	0.69 to 1.63	1.09	0.70 to 1.69	60	14.5	88	9.3	1.66	1.17 to 2.35	1.69	1.19 to 2.41
Polyols	51	14.4	13	8.4	1.83	0.97 to 3.48	1.91	1.01 to 3.65	25	6.0	31	3.3	1.90	1.11 to 3.26	1.95	1.14 to 3.36
Fructose	45	12.7	14	9.0	1.46	0.78 to 2.75	1.49	0.79 to 2.81	24	5.8	25	2.6	2.27	1.28 to 4.03	2.31	1.30 to 4.11

*Abbreviations: FODMAP* fermentable oligo-, di- and monosaccharides and polyols; *IBS* irritable bowel syndrome; *CI* confidence Interval; *OR* Odds ratio

^a^The question pertaining to these categories was: “If you react (to food), to what kind is that?”

^b^Adjusted for gender and age

^c^Assumed FODMAP content of the response(s) to the open-ended question about food

Perceived food intolerance for specific food categories in the two study groups (*Giardia* group vs. control group) was further analysed according to IBS status. Among respondents with IBS, the *Giardia* group reported vegetables, fruit, alcohol and the FODMAP subgroup polyols significantly more often than did controls. Among respondents without IBS, dairy products, fatty foods, vegetables, fruit, high FODMAP, and the FODMAP subgroups lactose, polyols and fructose were reported significantly more frequently by the *Giardia* exposed group than by controls (Table 4). The test for effect modification by IBS on perceived food intolerance was negative for these data.

We also investigated the difference in perceived food intolerance for the specific food categories between respondents with IBS compared to respondents without IBS when stratified according to exposure status. Within the exposed stratum respondents with IBS had statistically significantly more perceived intolerance for the food categories dairy products, spicy foods, fatty foods, vegetables, fruit, cereals and alcohol. Within the control stratum respondents with IBS had statistically significantly more perceived intolerance for the same food categories as mentioned above except for spicy foods, vegetables and alcohol (Additional file 1: Table S1).

Sub-group analyses on cases with moderate or severe symptoms from intake of food were of low value because of a low number of cases. No association was found between different subtypes of IBS (diarrhoea-predominant, obstipation-predominant, and mixed) and perceived food intolerances (Additional file 2: Table S2).

There was a tendency towards women reporting a higher prevalence of perceived food intolerance for most food categories than men, but this tendency was the same in both the exposed and the control group (Additional file 3: Table S3).

## Discussion

The main result of this study is that there was a higher prevalence of perceived food intolerance in the exposed group compared to a control group three years after verified *Giardia* infection. IBS was not an effect modifier for this association. Perceived intolerance to high FODMAP foods and low FODMAP foods were both statistically significantly associated with exposure to *Giardia* infection.

### Limitations and strengths

Some of the limitations regarding the data used in this study have been described before [4, 24]. The response rate in the exposed group (65,3 %) is reasonably high, however, selection bias cannot be ruled out. This may impact the prevalence, but estimates of association are more robust. The exposed group is selected on the basis of having seen a doctor and thus having had giardiasis diagnosed by positive stool samples. The differences in characteristics of this group compared to those who might have had giardiasis without seeking medical attention is not known. Unbiased baseline information about IBS, food intolerance, previous gastrointestinal infections or other illnesses is impossible to obtain, as this may be regarded as a natural, unplanned experiment. However, this problem of missing information is similar for the exposed group and the control group, and most of these factors are presumed to be equally distributed between the two groups prior to the giardiasis outbreak.

The exposed group has had a defined gastrointestinal illness that may lead to increased wariness of possible causes of their abdominal complaints, including food intolerance. Hence they might not actually be more susceptible to intolerance per se.

The response rate in the control group (31.4 %) is relatively low and there is a risk of selection bias. The prevalence of IBS in our control group is 14.0 %, which is a little higher than 8.4 %, the prevalence in the general Norwegian population as found in a large public health survey in 2006 [25]. Our study used the Rome III criteria, which have been shown in a study [26] to find a higher prevalence of IBS than the Rome II criteria used in the above-mentioned study. In sum, this may indicate that our control group is not too dissimilar from the general population. Again, the investigation of associations, with use of relative outcome measures such as OR, depends to a lesser degree on such biases.

The questionnaire items about food have not been validated, and the reliability is not known. They do not constitute a complete assessment of the respondents’ diet. The classification of an open-ended question may be subject to interpreter bias, and there is a potential for misclassification to a varying degree depending on the specific category. All food categories and how the answers were coded were accounted for in a codebook. Although quantitative analyses on qualitative data is not straight forward, one advantage of using an open-ended question instead of a closed-ended one is that it is unguided by any preconceived theory. The respondents were free to answer whichever type of food they perceived as giving symptoms. Also, there were 971 responses to the open-ended question, many of which were readily and unambiguously coded to meaningful food categories. This study was performed in 2007, before the concept of FODMAP-content in the diet was generally known, and the responses will not be biased by the recent interest in this diet. Based on these considerations we found that a quantitative approach was justified.

### Interpretation

Perceived food intolerance in a post-infectious setting has been scarcely investigated. Short-term lactose malabsorption after giardiasis has been described, but with contradictory findings [27, 28]. Fat malabsorption with steatorrhoea and diarrhoea can occur in chronic giardiasis, as can folate, B12 and vitamin A deficiency [27], but these are usually resolved with appropriate treatment. In our study the prevalence of perceived intolerance for both dairy products and fatty foods is relatively high, and significantly higher in the exposed than in the control group. Our study is not designed to investigate the mechanisms behind perceived food intolerance.

Recent studies and reviews have elucidated some of the mechanisms behind the development of PI-IBS after infective gastroenteritis [1]. Similar pathophysiologic mechanisms have also been found in sporadic IBS [9]. In this study we find a similar pattern of perceived food intolerance among *Giardia* exposed respondents with IBS (predominantly PI-IBS) and controls with IBS (sporadic IBS), but with a tendency, sometimes statistically significant, towards the exposed more often reporting intolerance for the specific food categories. Our results do not help clarify whether PI-IBS might be the same entity as sporadic IBS.

The prevalence of IBS and perceived food intolerance were measured at the same time. No inferences about causative pathways between IBS and perceived food intolerance can be made. We found that the exposed had a higher prevalence of perceived food intolerance than controls, and it has previously been found that this group has a higher prevalence of IBS [4]. There was also a significantly higher prevalence of perceived food intolerance among exposed in the no-IBS group. One hypothesis is that giardiasis causes alterations in the gastrointestinal tract that are important in the pathogenesis of both IBS and food intolerance. This does not suggest that the pathogenesis is identical, but there might be some common immunological pathways involved.

In our study 81.3 % of respondents with IBS in the exposed group and 78.4 % with IBS in the control group reported perceived food intolerance when this was defined as light, moderate or severe food-related abdominal complaints. Among respondents without IBS the proportions were 49.0 and 42.3 %, respectively. These results were comparable to a recent dietary survey performed on Irish IBS-patients (89.6 %) and a comparative group (55.0 %) [29]. The results for the non-IBS group is higher than what was found in a general UK population in 1994 (20.4 %) [30]. Other studies on IBS and perceived food intolerances have found prevalence ranging from 25 to 70 % [11, 19, 31, 32]. The reasons for the variance in prevalence of perceived food intolerance reported between studies might be due to different ways of measuring food intolerance, because of differences in the IBS-populations under investigation (e.g. inpatient vs. outpatient), and maybe due to a development in dietary trends over time.

Milk, dairy products, wheat products, caffeine, certain meat, certain vegetables, hot spices, alcohol, fat, fibre, fried food and smoked products are some of the foods stated in other studies to cause symptoms in IBS patients [10, 20]. The first nine of these food categories are also among the quantitatively most important in all investigated groups in our study, whereas the latter three are less frequently reported. Some of the above-mentioned food categories (dairy products, fatty foods and cereals) are significantly associated with IBS both in the exposed group and the control group. There are some similarities between the findings in our study and other studies on IBS and diet [10, 20]. However, as the validity and reliability of our questionnaire-items regarding food have not been tested, the results must be interpreted with caution.

There is a general tendency that the prevalence of perceived food intolerance for the various foods in our study is lower than that in other studies [11, 19, 29]. This could be due to the fact that most of the other studies use questionnaires with a predefined checklist of food items, which is known to overestimate the prevalence of intolerance to the included food items [33]. However, the prevalence of perceived food intolerance in our study is also lower than those reported in another study that used an open-ended question to map food perceived to cause symptoms [29]. This could be partially explained by a stricter coding of some of the food categories in our study, and also due to the fact that the patients included in the study were recruited from a gastroenterology clinic, and might thus be more severely ill than our respondents, who were recruited from the general population.

We found a statistically significant association between exposure status and perceived intolerance to both high and low FODMAP foods. Because the categories of high and low FODMAP foods were not mutually exclusive, the strength of the associations could not be compared statistically, but rather the results had to be interpreted more subjectively. The OR for high FODMAP foods was slightly higher than that for low FODMAP foods both in the unstratified and stratified (according to IBS-status) analyses, but with substantial overlap of the confidence intervals (Tables 3 and 4). The current study does not contradict or support the findings from other studies suggesting that high FODMAP content may add to symptoms among vulnerable individuals.

Food intolerance in IBS should be further investigated, especially with randomized controlled diet intervention studies in primary health care. We would propose that such a diet could be based on the FODMAP concept, but also include a tailor-made diet based on the patient’s perceived intolerances, followed by reintroduction.

## Conclusion

*Giardia* exposed participants had a higher prevalence of perceived food intolerance than a control group three years after acute gastroenteritis. The association between exposure to *Giardia* infection and perceived food intolerance differed between the IBS group and the no-IBS group, but IBS was not a significant effect modifier for the association. There was a significantly higher prevalence of perceived intolerance to foods both high and low in FODMAP content in the exposed group as compared to the control group. Our findings did not indicate a stronger association between *Giardia* exposure and perceived intolerance to high FODMAP foods as compared to low FODMAP foods.

## Additional files

Additional file 1: Table S1. Comparison of perceived food intolerance according to food categories and FODMAP content among Giardia exposed and a control group, stratified according to IBS status. 3 years after outbreak of giardiasis in Bergen, Norway, 2004. (DOCX 33 kb) Additional file 2: Table S2. Perceived food intolerance according to IBS subtype among Giardia exposed (*n* = 355) and controls (*n* = 155) with IBS 3 years after an outbreak of giardiasis in Bergen, Norway, 2004. (DOCX 36 kb) Additional file 3: Table S3. Comparison of 817 Giardia exposed and 1128 controls stratified according to gender, on perceived food intolerance in general and according to food categories and FODMAP content 3 years after outbreak of a Giardia-epidemic in Bergen, Norway, 2004. (DOCX 24 kb)
