# Supplementary material for: Perceived food intolerance and irritable bowel syndrome in a population 3 years after a giardiasis-outbreak: a historical cohort study
Source: BMC Gastroenterol. 2015 Nov 19;15:164. doi: 10.1186/s12876-015-0393-0 (PMC4653841; doi:10.1186/s12876-015-0393-0)
Supplement: Additional file 1: Table S1. — Comparison of perceived food intolerance according to food categories and FODMAP content among Giardia exposed and a control group, stratified according to IBS status. 3 years after outbreak of giardiasis in Bergen, Norway, 2004. (DOCX 33 kb) [file 12876_2015_393_MOESM1_ESM.docx]

| Table S1: Comparison of perceived food intolerance according to food categories and FODMAP content among *Giardia* exposed and a control group, stratified according to IBS status. 3 years after outbreak of giardiasis in Bergen, Norway, 2004. | | | | | | | | | | | | | | | | | | | | | | | |
| --- | --- | --- | --- | --- | --- | --- | --- | --- | --- | --- | --- | --- | --- | --- | --- | --- | --- | --- | --- | --- | --- | --- | --- |
|  | **Exposed  N=770** | | | | | | | | | | |  | **Controls N=1105** | | | | | | | | | | |
|  | **IBS  N=355** | |  | **No-IBS N=415** | |  | **Unadjusted** | |  | **Adjusted^b^** | |  | **IBS N=155** | |  | **No-IBS N=950** | |  | **Unadjusted** | |  | **Adjusted^b^** | |
| **Food categories***^a^* | **n** | **%** |  | **n** | **%** |  | **OR** | **95% CI** |  | **OR** | **95% CI** |  | **n** | **%** |  | **n** | **%** |  | **OR** | **95% CI** |  | **OR** | **95% CI** |
|  |  |  |  |  |  |  |  |  |  |  |  |  |  |  |  |  |  |  |  |  |  |  |  |
| *Food Categories* |  |  |  |  |  |  |  |  |  |  |  |  |  |  |  |  |  |  |  |  |  |  |  |
| Dairy products | 96 | 27.0 |  | 64 | 15.4 |  | 2.03 | 1.43 to 2.90 |  | 1.96 | 1.37 to 2.81 |  | 38 | 24.5 |  | 90 | 9.5 |  | 3.10 | 2.03 to 4.75 |  | 2.96 | 1.92 to 4.54 |
| Spicy foods | 69 | 19.4 |  | 48 | 11.6 |  | 1.85 | 1.24 to 2.75 |  | 1.77 | 1.18 to 2.65 |  | 26 | 16.8 |  | 109 | 11.5 |  | 1.56 | 0.98 to 2.48 |  | 1.48 | 0.93 to 2.37 |
| Fatty foods | 31 | 8.7 |  | 16 | 3.9 |  | 2.39 | 1.28 to 4.44 |  | 2.27 | 1.21 to 4.23 |  | 8 | 5.2 |  | 18 | 1.9 |  | 2.82 | 1.20 to 6.60 |  | 2.76 | 1.17 to 6.49 |
| Vegetables | 79 | 22.3 |  | 39 | 9.4 |  | 2.76 | 1.82 to 4.17 |  | 2.54 | 1.67 to 3.86 |  | 24 | 15.5 |  | 89 | 9.4 |  | 1.77 | 1.09 to 2.88 |  | 1.61 | 0.99 to 2.64 |
| Fruit | 49 | 13.8 |  | 25 | 6.0 |  | 2.50 | 1.51 to 4.14 |  | 2.37 | 1.42 to 3.95 |  | 12 | 7.7 |  | 31 | 3.3 |  | 2.49 | 1.25 to 4.96 |  | 2.28 | 1.14 to 4.57 |
| Cereals | 91 | 25.6 |  | 36 | 8.7 |  | 3.63 | 2.39 to 5.51 |  | 3.41 | 2.24 to 5.20 |  | 36 | 23.2 |  | 63 | 6.6 |  | 4.26 | 2.71 to 6.69 |  | 3.98 | 2.52 to 6.28 |
| Alcohol | 43 | 12.1 |  | 22 | 5.3 |  | 2.46 | 1.44 to 4.20 |  | 2.43 | 1.42 to 4.15 |  | 8 | 5.2 |  | 34 | 3.6 |  | 1.47 | 0.67 to 3.23 |  | 1.46 | 0.66 to 3.23 |
| Coffee | 23 | 6.5 |  | 16 | 3.9 |  | 1.73 | 0.90 to 3.32 |  | 1.73 | 0.90 to 3.35 |  | 12 | 7.7 |  | 40 | 4.2 |  | 1.91 | 0.98 to 3.73 |  | 1.90 | 0.97 to 3.72 |
| Soda | 9 | 2.5 |  | 7 | 1.7 |  | 1.52 | 0.56 to 4.11 |  | 1.46 | 0.54 to 3.98 |  | 6 | 3.9 |  | 17 | 1.8 |  | 2.21 | 0.86 to 5.70 |  | 2.11 | 0.82 to 5.45 |
|  |  |  |  |  |  |  |  |  |  |  |  |  |  |  |  |  |  |  |  |  |  |  |  |
| *FODMAP Content^c^* |  |  |  |  |  |  |  |  |  |  |  |  |  |  |  |  |  |  |  |  |  |  |  |
| High FODMAP | 186 | 52.4 |  | 116 | 28.0 |  | 2.84 | 2.10 to 3.83 |  | 2.72 | 2.01 to 3.69 |  | 74 | 47.7 |  | 201 | 21.2 |  | 3.40 | 2.40 to 4.84 |  | 3.24 | 2.27 to 4.62 |
| Low FODMAP | 145 | 40.8 |  | 83 | 20.0 |  | 2.76 | 2.01 to 3.81 |  | 2.66 | 1.92 to 3.67 |  | 57 | 36.8 |  | 171 | 18.0 |  | 2.65 | 1.84 to 3.82 |  | 2.56 | 1.77 to 3.69 |
|  |  |  |  |  |  |  |  |  |  |  |  |  |  |  |  |  |  |  |  |  |  |  |  |
| *FODMAP subtype* |  |  |  |  |  |  |  |  |  |  |  |  |  |  |  |  |  |  |  |  |  |  |  |
| Oligosaccharides | 131 | 36.9 |  | 58 | 14.0 |  | 3.60 | 2.53 to 5.12 |  | 3.39 | 2.37 to 4.85 |  | 51 | 32.9 |  | 135 | 14.2 |  | 2.96 | 2.02 to 4.34 |  | 2.73 | 1.85 to 4.03 |
| Lactose | 93 | 26.2 |  | 60 | 14.5 |  | 2.10 | 1.46 to 3.02 |  | 2.03 | 1.41 to 2.94 |  | 39 | 25.2 |  | 88 | 9.3 |  | 3.29 | 2.16 to 5.03 |  | 3.15 | 2.05 to 4.84 |
| Polyols | 51 | 14.4 |  | 25 | 6.0 |  | 2.62 | 1.59 to 4.32 |  | 2.50 | 1.51 to 4.14 |  | 13 | 8.4 |  | 31 | 3.3 |  | 2.71 | 1.39 to 5.31 |  | 2.55 | 1.30 to 5.00 |
| Fructose | 45 | 12.7 |  | 24 | 5.8 |  | 2.37 | 1.41 to 3.97 |  | 2.30 | 1.37 to 3.88 |  | 14 | 9.0 |  | 25 | 2.6 |  | 3.67 | 1.87 to 7.24 |  | 3.57 | 1.81 to 7.05 |
| *Abbreviations:* FODMAP: fermentable oligo-, di- and monosaccharides and polyols; IBS: irritable bowel syndrome; CI: confidence Interval; OR: Odds ratio.  a The question pertaining to these categories was: “If you react (to food), to what kind is that?”  b Adjusted for gender and age. c Assumed FODMAP content of the response(s) to an open-ended question about food. | | | | | | | | | | | | | | | | | | | | | | | |
