# Supplementary material for: Perceived food intolerance and irritable bowel syndrome in a population 3 years after a giardiasis-outbreak: a historical cohort study
Source: BMC Gastroenterol. 2015 Nov 19;15:164. doi: 10.1186/s12876-015-0393-0 (PMC4653841; doi:10.1186/s12876-015-0393-0)
Supplement: Additional file 2: Table S2. — Perceived food intolerance according to IBS subtype among Giardia exposed (n = 355) and controls (n = 155) with IBS 3 years after an outbreak of giardiasis in Bergen, Norway, 2004. (DOCX 36 kb) [file 12876_2015_393_MOESM2_ESM.docx]

| Table S2: Perceived food intolerance according to IBS subtype among *Giardia* exposed (n = 355) and controls (n = 155) with IBS three years after an outbreak of giardiasis in Bergen, Norway, 2004 | | | | | | | | | | | | | | | | |
| --- | --- | --- | --- | --- | --- | --- | --- | --- | --- | --- | --- | --- | --- | --- | --- | --- |
|  | | | | | | | | | | | | | | | | |
|  | Perceived food intolerance**^a,b^** | | | | | | | | | | | | | | | |
| **Group** |  | **Overall^a^** | |  |  | **Food category^b^** | | | | | | | | | | |
|  |  | **Yes^a^** | |  |  | **Dairy products** | |  | **Spicy foods** | |  | **High FODMAP** | |  | **Low FODMAP** | |
|  |  | **n** | **%** |  |  | **n** | **%** |  | **n** | **%** |  | **n** | **%** |  | **n** | **%** |
| IBS Constipation |  | 51 | 81.0 |  |  | 14 | 21.5 |  | 7 | 10.8 |  | 33 | 50.8 |  | 21 | 32.3 |
| IBS Diarrhoea |  | 150 | 81.1 |  |  | 47 | 24.9 |  | 37 | 19.6 |  | 96 | 50.8 |  | 79 | 41.8 |
| IBS Mixed |  | 171 | 81.4 |  |  | 60 | 28.2 |  | 47 | 22.1 |  | 111 | 52.1 |  | 88 | 41.3 |
| IBS Unsubtyped |  | 31 | 72.1 |  |  | 13 | 30.2 |  | 4 | 9.3 |  | 20 | 46.5 |  | 14 | 32.6 |
| P-value |  | 0.553 | |  |  | 0.640 | |  | 0.076 | |  | 0.928 | |  | 0.395 | |
| *Abbreviations:* FODMAP: fermentable oligo-, di- and monosaccharides and polyols; IBS: irritable bowel syndrome; P-value: p-value from Pearson’s chi square test (2-sided).  a The question pertaining to this category was: “Do certain types of food give you abdominal symptoms?” with four alternatives: none, light, moderate, severe, dichotomized to no (none) vs. yes (light, moderate or severe) b The question pertaining to these categories was: “If you react (to food), to what kind is that?” | | | | | | | | | | | | | | | | |
