# Supplementary material for: Perceived food intolerance and irritable bowel syndrome in a population 3 years after a giardiasis-outbreak: a historical cohort study
Source: BMC Gastroenterol. 2015 Nov 19;15:164. doi: 10.1186/s12876-015-0393-0 (PMC4653841; doi:10.1186/s12876-015-0393-0)
Supplement: Additional file 3: Table S3. — Comparison of 817 Giardia exposed and 1128 controls stratified according to gender, on perceived food intolerance in general and according to food categories and FODMAP content 3 years after outbreak of a Giardia-epidemic in Bergen, Norway, 2004. (DOCX 24 kb) [file 12876_2015_393_MOESM3_ESM.docx]

| Table S3: Comparison of 817 *Giardia* exposed and 1128 controls stratified according to gender, on perceived food intolerance in general and according to food categories and FODMAP content 3 years after outbreak of a Giardia-epidemic in Bergen, Norway, 2004. | | | | | | | | | | | | | | | | | |
| --- | --- | --- | --- | --- | --- | --- | --- | --- | --- | --- | --- | --- | --- | --- | --- | --- | --- |
|  | **Females N=1278** | | | | | | | |  | **Males N=667** | | | | | | | |
| **Perceived food intolerance** | **Exposed**  **N=540** | |  | **Controls N=738** | |  | **Unadjusted** | |  | **Exposed N=277** | |  | **Controls N=390** | |  | **Unadjusted** | |
|  | **n** | **%** |  | **n** | **%** |  | **OR^e^** | **95% CI** |  | **n** | **%** |  | **n** | **%** |  | **OR^e^** | **95% CI** |
|  |  |  |  |  |  |  |  |  |  |  |  |  |  |  |  |  |  |
| *Overall^a^* |  |  |  |  |  |  |  |  |  |  |  |  |  |  |  |  |  |
| Yes^b^ | 347 | 69.1 |  | 372 | 51.5 |  | 2.11 | 1.66 to 2.68 |  | 141 | 53.8 |  | 152 | 40.2 |  | 1.73 | 1.26 to 2.38 |
|  |  |  |  |  |  |  |  |  |  |  |  |  |  |  |  |  |  |
| *Food Categories^c^* |  |  |  |  |  |  |  |  |  |  |  |  |  |  |  |  |  |
| Dairy products | 127 | 23.5 |  | 104 | 14.1 |  | 1.88 | 1.41 to 2.50 |  | 36 | 13.0 |  | 25 | 6.4 |  | 2.18 | 1.28 to 3.73 |
| Spicy foods | 89 | 16.5 |  | 96 | 13.0 |  | 1.32 | 0.97 to 1.80 |  | 30 | 10.8 |  | 41 | 10.5 |  | 1.03 | 0.63 to 1.70 |
|  |  |  |  |  |  |  |  |  |  |  |  |  |  |  |  |  |  |
| *FODMAP Content^c,d^* |  |  |  |  |  |  |  |  |  |  |  |  |  |  |  |  |  |
| High FODMAP | 232 | 43.0 |  | 218 | 29.5 |  | 1.80 | 1.42 to 2.27 |  | 76 | 27.4 |  | 59 | 15.1 |  | 2.12 | 1.45 to 3.11 |
| Low FODMAP | 166 | 30.7 |  | 158 | 21.4 |  | 1.63 | 1.26 to 2.10 |  | 64 | 23.1 |  | 73 | 18.7 |  | 1.31 | 0.89 to 1.90 |
| *Abbreviations:* FODMAP: fermentable oligo-, di- and monosaccharides and polyols; IBS: irritable bowel syndrome; CI: confidence Interval; OR: Odds ratio.  a N=1224 for females (Exposed N=502, controls N=722) and N=640 for males (Exposed N=262, controls N=378) for this category.  b The question pertaining to this category was: “Do certain types of food give you abdominal symptoms?” with four alternatives: none, light, moderate, severe, dichotomized to no (none) vs. yes (light, moderate or severe) c The question pertaining to these categories was: “If you react (to food), to what kind is that?”  d Assumed FODMAP content of the response(s) to an open-ended question about food. e Breslow-Day test of homogeneity of the odds ratios between the two genders was negative for each category. | | | | | | | | | | | | | | | | | |
